# Temperamental traits of breath holding children: A case control study

**DOI:** 10.4103/0019-5545.43635

**Published:** 2008

**Authors:** A. Subbarayan, B. Ganesan, Anbumani, Jayanthini

**Affiliations:** Specialist Registrar in Psychiatry, Stratheden, Cupar, UK; 1 Specialist Registrar in Psychiatry, Nottingham, UK; 2 Assistant Professor, Child Guidance Clinic, ICH, Madras Medical College and RI, Chennai, India; 3 Professor of Psychiarty, Child Guidance Clinic, ICH, Madras Medical College and RI, Chennai, India

**Keywords:** Breath holding spells, temperament, children

## Abstract

**Background:**

Clinical observation and few anecdotal reports suggest that the children with breath holding spells (BHS) have certain temperamental traits, which predispose them to behave in certain way. They seem to have low frustration tolerance, which leads to adamant behavior. Vigorous crying, through various mechanisms, precipitates BHS.

**Materials and Methods:**

We assessed the temperamental traits of 30 children with BHS and compared them with 30 normal children after matching for age, sex, and socioeconomic status. Temperament was measured using ‘Temperament measurement Schedule’.

**Results:**

The data were analyzed using Mann-Whitney U test. The following temperamental traits, threshold of responsiveness (*P*<0.001), mood (*P*<0.001), activity level (*P*<0.001), intensity of emotions (*P*<0.001), distractibility (*P*<0.001) and rhythmicity (*P*<0.05) were found to be significantly different from the control group. Two factors, namely sociability (*P*<0.05) and energy level (*P*<0.001) were significantly higher in breath holders.

**Conclusions:**

Significantly differing temperamental traits in breath holders suggests that these could influence the behavioral pattern exhibited by them. Breath holding spells can act as an easy marker for difficult temperamental traits, which gives an early opportunity to shape their difficult behavior.

## INTRODUCTION

Breath holding spell (BHS), a stereo typed sequence of clinical events, is not an uncommon clinical presentation and is a frightening experience for the parents. During the spell, the child cries excessively because of frustration, pain, or both. At the end of prolonged expiration, they become apneic for several seconds and become either blue [cyanotic spells] or pale [pallid spells] and some children can experience both. Sometime they go in for tonic spasms and occasionally develop clonic movements.[1] A large epidemiological study in Karnataka, initiated by Indian Council of Medical Research, found that the prevalence of BHSs was 5.9% among 0 to 3 years age group, more common than other developmental disorders.[2]

Clinical observation over a period revealed that these children have certain temperamental characteristics, which predispose them to get frustrated easily and go in for adamant behavior. A study conducted in India showed that 56% of children with BHS exhibited excessive temper tantrums amongst other difficult behavior such as pica and head banging.[3] With this scenario, one can expect that parenting a child who exhibits BHS is likely to be more difficult than parenting a normal child. One study which assessed the stress on mothers with children exhibiting BHS concludes that the mothers develop disruptions in self image such as questioning their own parenting skills and experiencing their children as a less source of positive reinforcement.[4]

Temperament is a set of in-born traits that organize the child's approach to the world. They are instrumental in the development of the child's distinct personality. These traits also determine how the child goes about learning about the world around him/her. It is characteristic of an individual's nature, including susceptibilities to emotional stimulation, customary strength and speed of response, the quality of his/her prevailing mood and all the peculiarities of fluctuation and intensity of mood. These phenomenon are regarded as dependent on constitutional make up and therefore largely heredity in origin.[5] Though the temperament is an individual's inborn disposition, the subsequent course is determined by a complex interaction with the environment, which in turn is also affected by the dispositions.[6]

If these children with BHSs have difficult temperament, parents may need to adapt and tailor their parenting strategies to the temperamental characteristics of the child. These adapted parenting techniques may prove to be helpful in shaping these children's difficult behavior and this may reduce the stress on the parents.

### Aim

The primary purpose of this study was to explore the temperamental characteristics of the children with BHS by comparing with healthy children.

## MATERIALS AND METHODS

The sample included 60 children from the age group 1-4 years, and was divided into two groups.

1. Cases: This group consisted of 30 consecutive breath holders referred to Child Guidance Clinic during the study period.
  The inclusion criteria were:
  - Children within the age group of 1-4 years.
  - Children with BHSs for a minimum period of 3 months.
  The exclusion criteria were:
  - Any congenital heart disease.
  - Any CNS abnormalities.
  - Chronic medical illnesses.
2. Controls: This group consisted of 30 age and sex matched children belonging to same socioeconomic group as that of cases. They were selected from those attending out patient department of the same hospital for minor physical illnesses.

Place of study: Child Guidance Clinic, Institute of child health, Madras Medical College, Chennai.

Period of study: September and October, 2002.

Maneuver:

The written consent was obtained from all the parents of the 60 children after explaining about the study. The following instruments were applied to the parents of these children.

1. A semi structured proforma – this was compiled for the recording of socio demographic variables and the details about the BHSs.
2. Temperament measurement schedule (TMS).[7]

This schedule, devised by Savita Malhotra in 1995, was used to measure the temperament of the children. This is a standardized scale for Indian children. TMS was used after few modifications and translation into Tamil [local language] by two experienced child psychiatrists so that it suits for preschool children.

TMS measures nine temperament variables: approach-withdrawal, adaptability, threshold of responsiveness, mood, persistence, activity, intensity and distractibility. It consists of 45 items (five items in each of nine variables) rated on a five point scale. Definitions were provided for the two extreme scores one and five, with a mid point at three. The scores less than 3 were in negative direction and those more than three were in the positive direction. Mean scores for each of these variables were computed by dividing the total score by five.

The nine temperamental variables were reduced to five dimensions or factors.[7] Factor one, sociability consists of three variables, approach withdrawal, adaptability and threshold of responsiveness. Factor two consist of two variables namely mood and persistence. Factor three, energy level is constituted by activity level and intensity of reactions. Factor four consists of one variable, distractibility. Factor five also made of one variable, rhythmicity.

The mother was interviewed by giving adequate time in each case, which included assessment using the semi-structured proforma and the aforementioned TMS instrument. It took 30-40 min to complete the interview and assessing the temperament. The scores in the two groups were compared by Mann-Whitney U test, a non-parametric test, using SPSS version 11.

## RESULTS

There were 17 male and 13 female children in each group. In breath holders group, the youngest was of 12 months and eldest was aged 46 months. Majority of them (56.7%, *n* =17) were in the 12 to 24 months age range. Only four children were in 36 to 48 months age range and the rest (30%, *n* = 9) were aged between 24 and 36 months.

Among the cases, 40% of them had the onset of BHSs within first six months of their life, 23.3% of them in between 7 and 12 months of age and 26.7% first experienced between 13 and 24 months. The onset of BHS after two years of age, constituted only 10%. We categorized these children into three groups based on the frequency, whether they experienced the spells on daily, weekly, or monthly basis. Majority fell into ‘weekly group’ with 40% (*n* = 12), followed by ‘monthly group’ with 33.3% (*n* = 10). ‘Daily BHSs’ category constituted 26.7% of cases.

Among cases, only one child had hemoglobin (Hb) value above 11 g%. For three children, value was not available and the rest, had ‘Hb’ levels in anemic range. Majority of the breath holders (88.9%) had Hb level between 7 and 11 g%, while 7.4% of cases showed Hb level less than 7 g. All the cases were on iron therapy, a common and recognized treatment for BH spells.

As seen in Table 1, the children in the index group (breath holders) showed low threshold of responsiveness while comparing with the control group and the difference is statistically highly significant with the *P* value <0.001. This was also reflected in the sociability dimension, which is factor one, with *P* value <0.05, expressing the sensitive nature of the children.

**Table 1:** Results – Temperamental traits of Cases and controls

	Mean rank	Mann Whitney u	Z(negative)	*P* value
Approach – withdrawal				
Case	29.45	418.500	.427	.637
Control	31.55			
Adaptability				
Case	30.93	437.000	.195	.846
Control	30.07			
Threshold of responsiveness				
Case	20.70	156.000	4.375	<.001
Control	40.30			
Mood				
Case	23.40	237.000	3.188	<.005
Control	37.60			
Persistence				
Case	33.95	346.500	1.542	.123
Control	27.05			
Activity level				
Case	37.70	234.000	3.233	<.005
Control	23.30			
Intensity of reactions				
Case	43.37	64.000	5.749	<.001
Control	17.63			
Distractibility				
Case	22.75	217.500	3.472	<.005
Control	38.25			
Rhythmicity				
Case	25.10	288.000	2.411	<.05
Control	35.90			
Sociability				
Case	24.17	260.000	2.818	<.05
Control	36.83			
Emotionality				
Case	27.97	374.000	1.130	.259
Control	33.03			
Energy				
Case	42.32	95.500	5.255	<.001
Control	18.68			

The breath holders tend to be angry, annoyed, and irritable and discontented by scoring low in the variable ‘Mood’ than the control group. This difference achieved was statistically significant.

The energy dimension score was high for the index group as they scored high on constituent variables, ‘activity level’ and ‘intensity of reactions’, reflecting their high psychic and physical energy in behavior and reactions to the environmental stimuli. High scores in the ‘energy’ dimension and the ‘intensity of reactions’ are highly significant (*P*<0.001), where as the high score on the temperamental variable ‘activity level’ is significant (*P*<0.05).

The index group significantly scored less on the fourth factor, which contains only one temperament variable ‘distractibility’. It means that it may be difficult to humor or ease them while they are in bad mood, unlike normal children. Also in the other way, they were found to be less distractible if they were interested in a particular activity. The children belonging to the control group were well-regulated one by scoring high on ‘Rhythmicity’ dimension when compared to index group. The daily routines like sleeping and eating were irregular in cases (*P*<0.05).

The statistical analysis showed that no association could be made out between frequency of spells, age of onset of spells and hemoglobin level with the temperamental variables of the index group.

## DISCUSSION

As discussed earlier, ‘temperament’ refers to the characteristic mood, activity, reactivity and emotionality of the individual. Many studies have shown that infants have considerable variation in the temperament.[8,9] In addition, the individual specific reaction pattern appears in the first few months of life, persists in a stable form there after, and significantly influences the nature of the child's response to all environmental events, including childcare practices.[10]

In our study, we found that the breath holders were very sensitive in nature. These children's mothers reported that their children were easily bothered and annoyed by noise and to light push from other children during play, reflecting the ‘low threshold of responsiveness’ to unpleasant incidents. It made them less sociable than the children without BHS, who tended to take things easily or ignored any unpleasant events. Interestingly, breath holders showed no difference to healthy children, in the ‘approach withdrawal’ and ‘adaptability’ and they behaved similarly to control group while approaching strangers and mingling with other children. In another study, researchers showed that 88% of the breath holders had ‘frustration or anger’ as the initiating factor for BHS.[3] One can also argue that these two factors simply reflect partly the underlying temperament of the children exhibiting BHS. The proneness to be frustrated easily, in turn reflects the high sensitivity or low threshold of tolerance of the breath holders, indirectly confirming our findings.

Mothers of children with BHS frequently found them to be annoyed, discontented and easy to get anger, a state of negative emotional state. This was noted by mothers as frequent fights and or arguments with other children and being annoyed. On the contrary, most of the time normal children avoided fighting or indulging in frequent conflicts with other playmates and remained disturbed only for a short while if their wish was not fulfilled.

Breath holders having scored significantly low score in ‘distractibility’ dimension took a long time to come out of any negative emotional state even when consoled by their mothers. This of course made their parents to take dramatic measures to please them, further blurring the boundaries. Interestingly, this study found that if the breath holders indulged in any interesting activity or play, they would not be easily diverted away and tended to ignore the call from their mothers. Normal children could be easily drawn away from their bad mood and they responded better to the calls of mother while playing in comparison to the breath holders. So, we speculate that children with BHSs have a ‘tendency to stay’ in their peak emotional states. The children without BHS were found to be well regulated in food habits, sleep pattern and bowel movements making the caregiver's job easier. On the contrary, the breath holders exhibited poor ‘rhythmicity’ in all these biological functions making it a bit difficult to caregivers.

The results of this study also suggest that breath holders are ‘high active’ with the motor component of any activity being more intense than the control group. They were frequently described as ‘on the run constantly’. The scoring on ‘Intensity of reaction’ was higher than the normal children and made them to enjoy and express themselves well in case of positive environment. At the same time, their intensity of negative reactions like expressing anger, crying and getting upset was also high in case of disappointment, adding stress on the part of mothers.

Our findings in this study are mostly in line with another study, where the researchers found the children with BHSs to differ significantly on distractibility/hyperactivity, adaptability and demandingness, though they had used a different scale (Parent Stress Index). The first domain of this scale assessed the child characteristics and the role of the child's temperament and behavior.[4] Interestingly, another study, did not find any significant differences in behavioral profiles between children with BHS and control children.[11]

### Clinical implications

Our study has clearly shown that breath holders are different from other children, being more sensitive, reacting dramatically and intensely to any negative environment and with poor rhythm in biological functions. As we know well that child rearing is an active process, involving the active participation of both the parties, continuously shaping the attachment in between. Our findings suggest that it is more likely that the child rearing experience of parents of children with BHS, might not be as easy as the experience of parents with normal children. In Indian culture, in most families mother being a primary carer, has more responsibilities and expectations in the upbringing of children. In this background, one can guess it puts mothers under stress. This may cause them to develop dysfunctional parenting style in an effort ‘to avoid triggering BHS and difficult behavior’. For example, they may easily yield into their children's unreasonable demands, rather than setting boundaries. For the children with BHS, this frequent indulging in ‘immediate gratification’, further would nourish their impatience and adamant behavior, and reduce the opportunity to develop positive adaptive skills and shaping their temperament.

It is important to educate the mothers that BHS is a benign medical condition, which may empower them to apply firm boundaries, rather than switching to anxiety state, blurring the boundaries and further reinforcing children's behavioral problems. Professionals, whenever possible should teach them, simple behavioral techniques such as positive reinforcement of desired behavior and avoiding being overtly attentive when these children exhibited adamant behavior. All other adults in the family should be encouraged to have a uniform approach, for the behavioral approach to be more effective and long lasting.

Further research in this area focusing on the persistence of the difficult temperament into adolescence and adult life would be useful. If that is the case, it would be interesting to study the effects of early behavioral interventions on the later life of children with BHS.

### Limitations of the study

1. Temperament Measurement Schedule was used after certain modifications (originally designed to measure the temperament of children between 4-14 years) as there was no Indian scale available for preschool children. Nevertheless, these minimal changes were made by two experienced child psychiatrists to make it applicable to preschool age group and further finer adjustments were made after a pilot study. This might have improved the validity of assessments.
2. There was no blinding in this study.

## References

[1] DiMario FJ (2001). Prospective study of children with cyanotic and pallid breath holding spells. Paediatrics.

[2] Srinath S, Girimaji SC, Gururaj G, Seshadri S, Subbakrishna DK, Bhola P (2005). Epidemiological study of child and adolescent psychiatric disorders in urban and rural areas of Bangalore, India. Indian J Med Res.

[3] Bhatia MS, Singhal PK, Dhar NK, Nigam VR, Malik SC, Mullick DN (1990). Breath holding spells; Analysis of 50 cases. Indian Paediatr.

[4] Mattie-Luksic M, Javornisky G, DiMario FJ (2000). Assessment of stress in mothers of children with severe breath-holding spells. Pediatrics.

[5] David HP, Bracken Von H, Allport (1961). Europian and American Theories of Personality. Perspectives in Personality Theory.

[6] Buss AH, Plomin R (1975). A temperament theory of personality development.

[7] Malhotra S (2002). Child Psychiatry in India.

[8] Stifer CA, Fox NA (1990). Infant reactivity: Physiological correlates of newborn and 5- month temperament. Dev Psychol.

[9] Worobey J, Blajda VM (1989). Temperament ratings at 2 weeks, 2 months, and 1 year: Differential stability of activity and emotionality. Dev Psychol.

[10] Caspi A, Silva PA (1995). Temperamental qualities at age three predict personality traits in young adulthood: Longitudinal evidence from a birth cohort. Child Dev.

[11] DiMario FJ, Burleson JA (1993). Behavior profile of children with severe breath-holding spells. J Pediatr.

